# A SICE (Società Italiana di Chirurgia Endoscopica e Nuove Tecnologie) observational prospective multicenter study on anatomical variants of the superior mesenteric artery: intraoperative analysis during laparoscopic right hemicolectomy—CoDIG 2 database (ColonDx Italian Group)

**DOI:** 10.1007/s13304-024-01787-6

**Published:** 2024-03-25

**Authors:** G. Anania, A. Campagnaro, M. Chiozza, J. Randolph, G. Resta, S. Marino, S. Pedon, A. Agrusa, D. Cuccurullo, R. Cirocchi, SICE CoDIG (ColonDx Italian Group)

**Affiliations:** 1 Unit of General Surgery, S. Anna University Hospital of Ferrara, Via Aldo Moro 8, Cona, FE Italy; 2 Department of Medical Science, University of Ferrara, Via Fossato di Mortara 64/B, 44121 Ferrara, FE Italy; 3 Georgia Baptist College of Nursing, Mercer University, Atlanta, GA 30341 USA; 4 Department of Surgical, Oncological and Oral Sciences, University of Palermo, Palermo, PA Italy; 5 Division of Laparoscopic and Robotic Surgery Unit, A.O.R.N. Colli Monaldi Hospital, Napoli, NA Italy; 6 Department of Medicine and Surgery, University of Perugia, Piazza Università 1, 06123 Perugia, PG Italy; 7 Division of Digestive and Emergency Surgery, Santa Maria Hospital, Via Tristano di Joannuccio 05100, Terni, TR Italy

**Keywords:** Vascular anatomy, Right colic artery, Superior mesenteric artery, Yada classification, Right hemicolectomy, Laparoscopy

## Abstract

Colorectal cancer, the third most common cancer worldwide, affects 40–45% of patients on the right side. Surgery, especially minimally invasive methods such as laparoscopic and robotic procedures, is the preferred treatment. However, these techniques present technical complications. The anatomical complexity and variations in vessel branching patterns pose challenges, particularly for less experienced surgeons. The CoDIG 2 is a nationwide observational study involving 76 specialized Italian general surgery departments focused on colorectal surgery. The centres were directed to maintain their standard surgical and clinical practices. The aim of this study was to analyse the intraoperative vascular anatomy of Italian patients who underwent laparoscopic right colectomy and explore the ligature techniques used by Italian surgeons. Surgeons reported information about vascularization of the right colon for 616 patients and about surgical anatomy of RCA for 368 patients. Fifty-three patients (10.8%) showed no RCA intraoperatively. The right colic artery (RCA) was categorized according to the Yada classification (types 1–4) during evaluation, and intraoperative assessments revealed that Yada type 1 was the most common type (55.2%), while radiologic evaluations revealed a higher prevalence of type 2. Furthermore, compared with the superior mesenteric vein (SMV), the RCA is more often located anteriorly according to intraoperative and contrast-enhanced CT examination; 59.9% were found in the anterior position during intraoperative examination, while 40.1% were found in the same position on preoperative contrast-enhanced CT. Vascularization of the right colon, including missing branches, additional branches, shared trunks, and retro-superior courses of the mesenteric vein, exhibited notable variations. To understand vascular variations, a preoperative radiological study is necessary; although there was no concordance between the intraoperative and radiological evaluations, this is a limitation of preinterventional radiological evaluation (PII) because it is always needed for oncological staging. This approach is especially critical for inexperienced surgeons to avoid potential complications, such as problematic bleeding.

## Introduction

According to the estimated 2020 incidence in the Global Cancer Observatory database (GLOBOCAN), colorectal cancer is the third most common cancer in the world, with 1.931.590 new cases (10%), and the second most common cause of death, with 935.173 deaths (9.4%) [1]. An analysis of the Surveillance, Epidemiology, and End Results (SEER) database revealed that 40–45% of all colon-rectal cancers are located on the right side [2, 3] .

During right colectomy, surgeons usually search for anatomical variations in vessels in common clinical practice to attempt correct central vessel ligation [4, 5]. Currently, laparoscopic identification is easier than intraoperative identification [6, 7]. In high-volume centres, this technical step is a requirement [8, 9]; in fact, correct central vessel ligation is associated with a high number lymph node dissections. For these reasons, some surgical groups aim to standardize the technique for right-sided colon cancer [10, 11].

In some cases, anatomical complexity and anatomical variations in vessel branching patterns pose challenges for young surgeons during laparoscopic or robotic right hemicolectomy [12, 13]. Consequently, accurate knowledge of possible anatomical variations is very important for safe oncological identification and dissection of vessels in the right colon [14, 15].

The aim of our study was to analyse the anatomical variations in the vessels of Italian patients who underwent laparoscopic right colectomy; we hope that the findings of our study will be added to the literature to improve the standardization of colorectal surgical procedures.

## Methods/design

The CoDIG2 (ColonDxItalian Group – Italian Right Colon Group) study (ClinicalTrials.gov; ID: NCT05943951) was a 6-month multicentre prospective observational study (01/04/2022 to 30/09/2022) concerning “laparoscopic right hemicolectomy” for cancer treatment. The aim of the present study was to report the steps Italian surgeons perform during “laparoscopic right hemicolectomy”, namely, ligature of vessels and extension of lymphadenectomy. Furthermore, a section of the study is dedicated to presenting intraoperative data about the anatomical variability of the vascularization of the right colon. Regarding the lymphadenectomy, our working group has recently published an article on the subject [16].

The CoDIG2 was designed based on relevant guidelines and regulations applicable to research involving human subjects, such as the World Medical Association Declaration of Helsinki and the Medical Research Involving Human Subjects Act. Written informed consent was obtained from all participants.

For this study, a prospective patient questionnaire was administered at the University of Ferrara and was submitted to all members of the SICE (Società Italiana di Chirurgia Endoscopica e Nuove Tecnologie).

### Outcomes

Anatomical variants of the superior mesenteric artery were categorized into types according to Yada’s classification [17].

The principal outcome was the absence of the right colic artery (RCA) (reported as type 4 according to the Yada classification) on evaluation during surgical dissection.

**Fig. 1:**
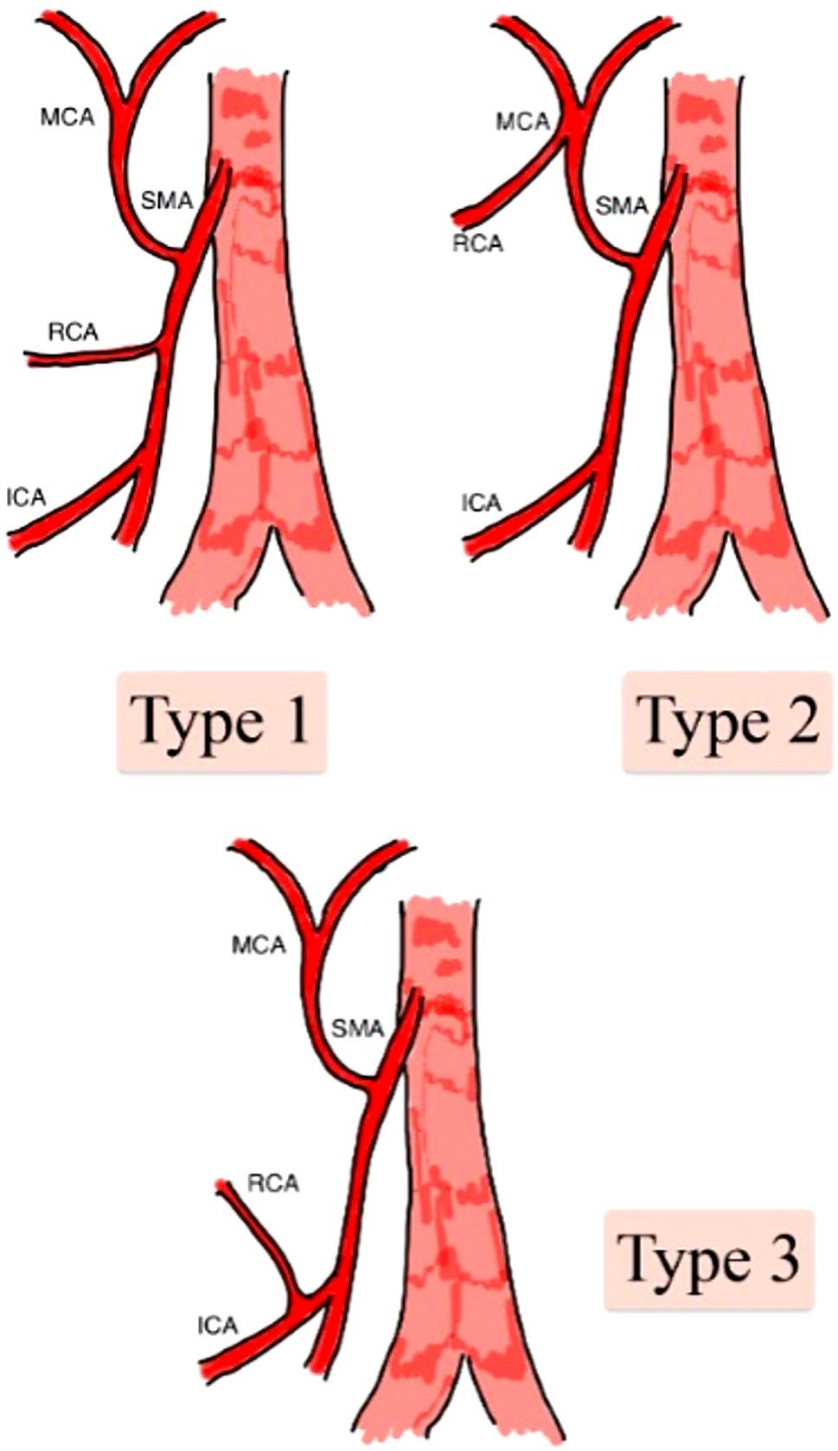
Yada classification of RCA variations. *SMA* superior mesenteric artery, *MCA* middle colic artery, *RCA* right colic artery, *ICA* ileocolic artery

**Fig. 2:**
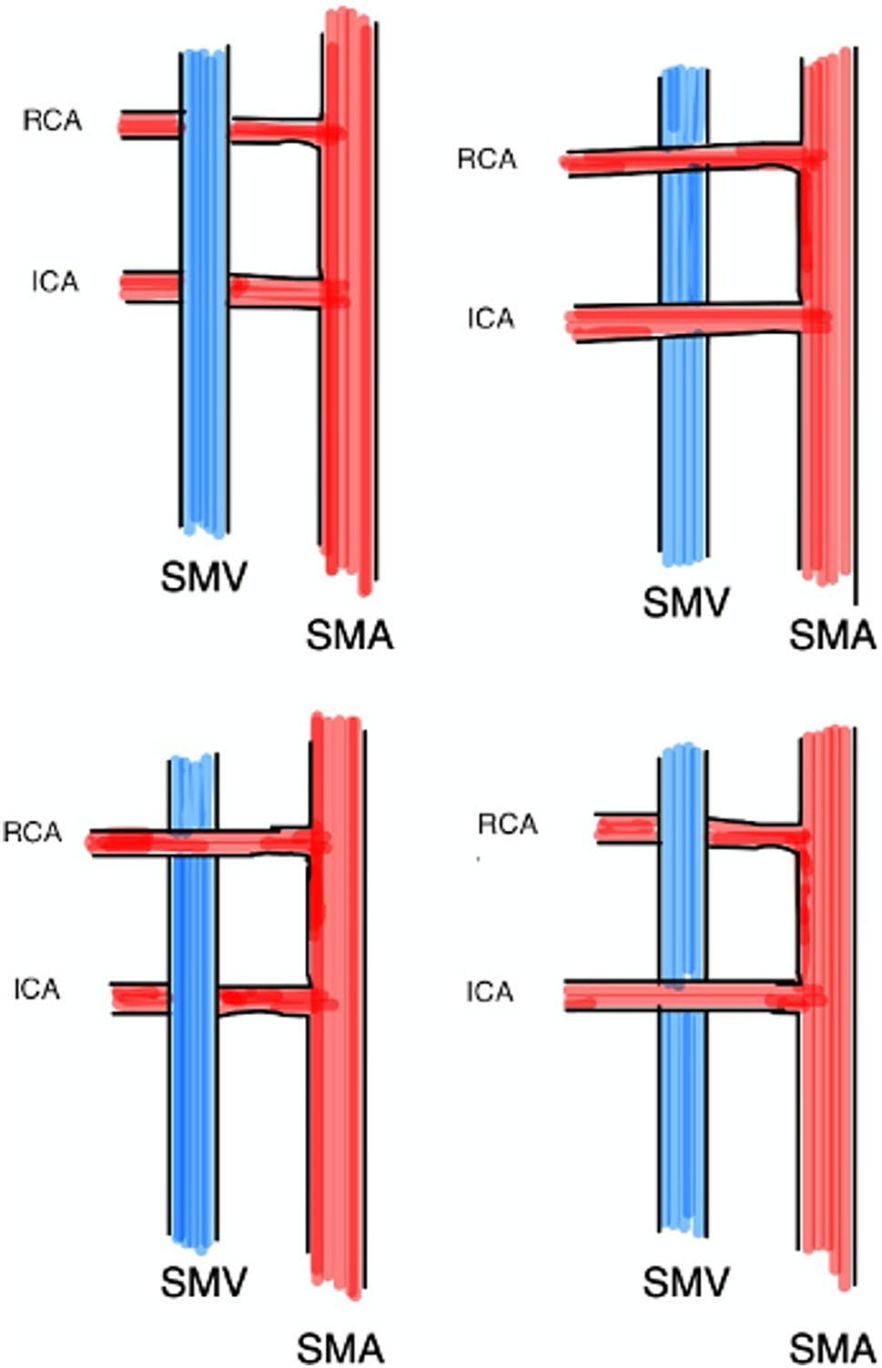
Topographic relationship between the RCA and SMV

The secondary outcomes were:

1. The RCA was categorized according to the Yada classification (Fig. 1) on evaluation during surgical dissection and on preinterventional (PII) radiological evaluation via computed tomography (CT):
  The RCA arises independently from the superior mesenteric artery (SMA) (Yada Type 1).
  The RCA and middle colic artery (MCA) have a common trunk (Yada Type.
  The RCA and ileocolic artery (ICA) have a common trunk (Yada Type 3).
2. Topographic relationship between the RCA and SMV on evaluation during surgical dissection and, on preinterventional (PII) radiological evaluation, the RCA was located anterior or posterior to the SMV (Fig. 2).

### Data collection

A member of each participating centre prospectively enrolled data for consecutive patients on a website sponsored by SICE for smart access through personal computers, tablets and smartphones. Consequently, we collected baseline data (sex, age, BMI, Charlson comorbidity index (BMI), American Society of Anaesthesiologists (ASA) score, preoperative diagnosis, TNM stage, histologic diagnosis, surgical technique, operative time, type of anastomosis, intraoperative complications, tumour-free margin, terminal ileum length, vascular ligature, mesocolon sail integrity and area, total lymph nodes/positive lymph nodes, ERAS protocol adherence, length of postoperative hospital stay, and postoperative complications) from the hospital electronic medical records system in our electronic database. A consultant recognized the vessel anatomy intraoperatively; subsequently, the same expert surgeons completed the questionnaire.

The inclusion criteria were as follows: all consecutive patients aged >18 years who underwent elective laparoscopic or robotic right colectomy. The exclusion criteria were emergency surgery, laparotomic right colectomy, ASA > IV, and pregnancy.

All patients underwent standard laparoscopic or robotic right colectomy with D2 lymphadenectomy.

In the PII evaluation, all patients underwent dynamic CT, and a senior radiologist reviewed the contrast-enhanced images.

### Statistical methods

Frequencies and crosstabulations were conducted with SPSS 27.0 software for artery location (anterior or posterior), evaluation type (intraoperative or contrast-enhanced CT), and Yada classification (Type 1 through Type 4). Bootstrap 95% confidence intervals were computed using 1000 bootstrapped samples. Following the recommendations of the American Statistical Association, we report 95% confidence intervals instead of *p* values from null hypothesis tests. Differences in *N* between analyses were due to patients with missing data for the variables being investigated [18].

## Results

In the CoDIG2 cohort, 788 patients were enrolled from 76 participating centres over a period of 6 months from April 2022 to October 2022; surgeons reported the vascularization of the right colon for only 368 patients. All patients were Caucasian (100%).

**Fig. 3:**
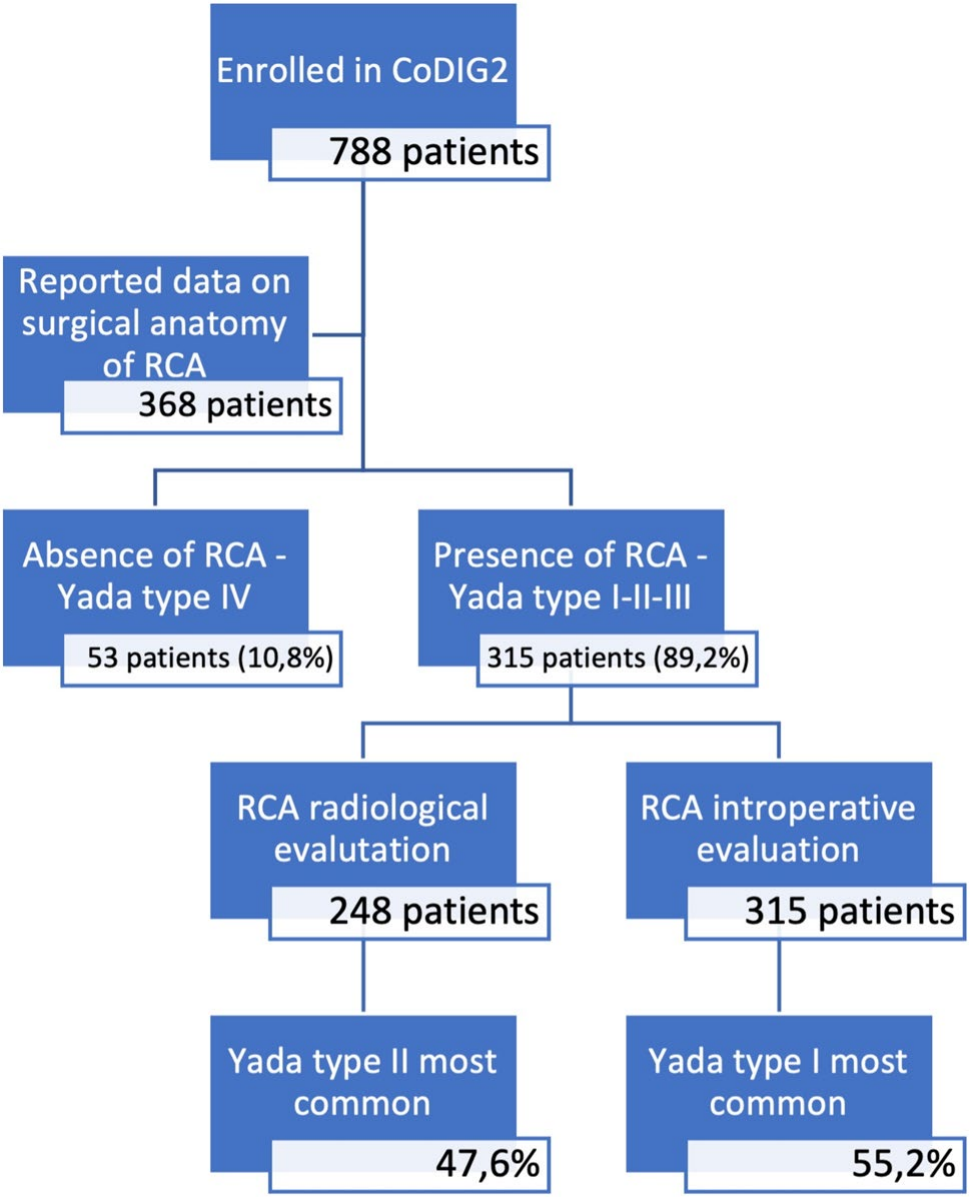
Absence of the RCA

Fifty-three patients had no RCA intraoperatively (10.8%), which is reported as type 4 according to the Yada classification (Fig. 3).

For the other 315 patients (89.2%), vascularization of the right colon artery was categorized through the Yada classification and disaggregated by evaluation type. Among the same patients, radiological and intraoperative findings were reported for 248 patients and 315 patients, respectively.

This analysis revealed that intraoperatively, Type 1 was the most common condition (55.2%). In contrast, radiological preoperative evaluation (contrast-enhanced CT) revealed that Type 2 was most common (47.6%) (Table 1).

**Table 1:** Frequencies and percentages of arterial vascularization from the superior mesenteric artery based on evaluation type and Yada’s classification

Evaluation type	Yada classification	Frequency	%	95% CI lower %	95% CI upper %
**Intraoperative**	**Type 1**	174	**55.2**	49.5	60.3
	Type 2	112	35.6	30.5	41.0
	Type 3	29	9.2	6.3	12.4
	Total	315	100.0	100.0	100.0
**Contrast-enhanced CT**	Type 1	99	39.9	33.9	46.0
	**Type 2**	118	**47.6**	41.5	54.0
	Type 3	31	12.5	8.5	16.9
	Total	248	100.0	100.0	100.0

*The RCA arises independently from the SMA (superior mesenteric artery) (Yada type 1).* This variation was the most common finding during intraoperative dissection (55.2%), but the rate was lower during radiological preoperative evaluation (contrast-enhanced CT) (33.9%). In type 1 patients, the RCA was located in the anterior position to the superior mesenteric vein (SMV) (55.04% of the patients).

*Common trunk of the RCA and MCA (middle colic artery) (Yada type 2).* This variation was the most common finding during radiological preoperative evaluation (contrast-enhanced CT) (47.6%), but the rate was lower for intraoperative dissection (35.6%). Among those classified as type 2, the common trunk anterior to the SMV was the most common location (66.39%).

*Common trunk of the RCA and ICA (ileo-colic artery) (Yada type 3).* This variation was the most uncommon, and the rates of radiological preoperative evaluation (contrast-enhanced CT) (12.5%) and intraoperative dissection (9.2%) were the same. In type 3 patients, the posterior position to the SMV was the most common location of the common trunk (66.39%).

Furthermore, the topographic location between the RCA and SMV (Figure 2) was evaluated intraoperatively in 315 patients, and it was evaluated preoperatively via contrast-enhanced CT in 248 patients. The evaluation of tumour type stratified by artery location revealed a different trend; in fact, for the intraoperative and contrast-enhanced CT images, 59.9% of the intraoperative cases were located in the anterior position, while 40.1% were located in the same position on preoperative contrast-enhanced CT images (Table 2).

**Table 2.** Frequencies and percentages of the different types of superior mesenteric arteries according to evaluation type and Yada classification

Artery location	Evaluation type	Frequency	%	95% CI lower %	95% CI upper %
Anterior	Intraoperative	190	59.9	54.7	65.0
	Contrast-enhanced CT	127	40.1	35.0	45.3
	Total	317	100.0	100.0	100.0
Posterior	Intraoperative	125	50.8	44.5	57.7
	Contrast-enhanced CT	121	49.2	42.3	55.5
	Total	246	100.0	100.0	100.0

## Discussion

In this prospective observational study, we reported the results of vascular variations during intraoperative and radiological evaluations performed on patients enrolled in CODIG 2 who underwent laparoscopic right colectomy.

Currently, laparoscopic and robotic evaluations routinely reveal anatomical details that were not commonly visualized; consequently, there is increasing knowledge of vascular variations.

The evaluation of these anatomical vascular variants is a basic step for the standardization of oncological right hemicolectomy. In fact, these new anatomical data have thus made this technique safer because it is associated with a reduction in postoperative morbidity related to ligatures [14].

In addition, the presence of arterial variants seems to be associated with better outcomes in terms of oncological radicality; recently, Efetov et al. reported the results of a large comparative study of 260 Russian and Chinese patients. They suggested that in Western countries, increased complexity in performing D3 lymph node dissection is associated with an increased prevalence of surgically challenging anatomic variations in the right colon vessels [19].

Currently, CODIG 2 is the largest study in the literature in which an intraoperative description of the arteries of the right colon has been reported; to date, only eight articles have been published on the topic, and they have included a total of 930 patients [13, 20–26].

Furthermore, in our study, anatomical evaluation was always performed during laparoscopic right hemicolectomy; only Wu [20], Ohsawa [25] and Lee [22] reported performing laparoscopic evaluation during right hemicolectomy, but the numbers of patients were lower 60, 205 and 116, respectively. Furthermore, Alsabilah reported performing a laparoscopic evaluation but did not differentiate the number of patients examined laparoscopically or via open approaches [13]. All the studies in which anatomical analysis was performed were conducted in Asia (Korea, Japan and China); however, the data in our study were based only on the Italian population.

Our results do not agree with the findings of an extensive systematic review of international literature based on 41 studies (n = 4691 patients) [27].

Regarding the absence of an RCA, our results (absence in 10.4% of patients) differed from those of the aforementioned systematic review and meta-analysis, which reported absence in 27.4% of patients [27]; this very obvious difference was also present in laparoscopic studies conducted in Asia (Wu 45%, Ohsawa 48.8%, Lee 67.3%) [20, 22, 25], which could suggest that ethnic factors may be the basis of this difference. In addition, the origin of the RCA was evaluated and categorized according to the Yada classification:

- *Yada type 1* (an RCA that originates independently of the SMA) was the most common type according to the intraoperative evaluation in our study (55.2%) and in the systematic review, where the percentage was greater (68.9%).
- *Yada type 2* (common branch for the RCA and MCA) was the second variant by frequency according to the intraoperative analysis of our study (35.6%) and systematic review, where the percentage was lower (17.7%).
- *Yada type 3* (common branch for the RCA and ICA) was the rarest variant according to the intraoperative evaluation (9.2%) and the systematic review (13.2%).

In patients with anatomical variations of the RCA, there is a great surgical risk of not recognizing a type 2 or 3 Yada variant; this is of great surgical importance because, in some cases, the MCA can be confused with the RCA and cut/ligated. This could impair the blood supply to the distal transverse colon [28].

For these reasons, the importance of a preoperative radiological study has been supported by numerous surgeons [29], who argue that right colon vascularization can be delineated radiologically, and that preoperative vascular mapping is a necessary component of the CME technique.

In addition, another critical point related to the anatomical variants of the vascularization of the right colon is the correct understanding of the relationship between the SMV and the branches of the SMA. Knowledge of such topographic reports is of paramount importance for preventing iatrogenic lesions of the SMV, which are serious and potentially fatal during vascular dissection of right hemicolectomies [30].

This condition represents a very important risk because these topographic relationships are highly variable. In our study, 59.9% of variants located in the anterior course of the RCA were found via intraoperative evaluation, whereas 40.1% were found via preoperative contrast-enhanced CT analyses (Table 2). This result is not in line with the literature in which the RCA precedes the SMV in 53.04% of patients.

The learning curve of laparoscopic and robotic techniques for right colectomy was evaluated. A risk-adjusted cumulative sum (CUSUM) model was used from Tekkis et al. to evaluate the learning curve for right colon cancer treatment; this study reported that 55 patients were needed to prove proficiency for right colectomy. During the learning curve, the most common mistake is dissection of the wrong surgical layers, which is common in the deep retroperitoneal space, especially for a lesion in Gerota's fascia or an injury to deep retroperitoneal structures [31, 32].

Identification and central ligation of the main mesocolic vessels can be difficult for novice surgeons; for this reason, several surgeons have suggested the usefulness of three-dimensional printing of superior mesenteric vessels for determining the course of the superior mesenteric vessels [33, 34].

In fact, right colectomies performed by novice surgeons are commonly prolonged procedures with increased bleeding and decreased lymph node resection [34].

This study is limited by the difficulty of identifying vascular anatomy during laparoscopic or robotic surgery. According to Kuzu et al., it is practically impossible to trace each vessel to its origin in real-time surgery, especially within the fatty mesocolon, thereby increasing the risk of severe complications. Additionally, it is challenging to ascertain the names of each tributary of the superior mesenteric artery (SMA) and superior mesenteric vein (SMV) until all tributaries are centrally ligated and when the specimen is removed [15].

Nonetheless, our study has several limitations, most of which involved subjective evaluation by surgeons or radiologists. In fact, differences in anatomical knowledge for surgical proficiency can lead to differences in opinions about the vessels. For this reason, SICE has a new ongoing study in which the researchers aim to reduce this bias [36, 37]:

- The surgeon photographed the vessels during intraoperative dissection and sent the photos to three consultants to check the correctness of the identification.
- The radiologist extracted the CT photogram and sent the images to three consultants to check the correctness of the identification.

Accurate identification of anatomical structures involved in surgical intervention is a crucial component of the undergraduate medical curriculum and frequently difficult, as time constraints often limit the depth of instruction. [37]. We hope that this new research can resolve this “information gap” for right minimally invasive colectomy regarding poor knowledge of vascular variants. In fact, vascular variations are still the cause of bias in surgical training that cannot be resolved from surgical anatomy dissection courses [38].

## Conclusion

Right colectomy performed with minimally invasive surgical techniques (laparoscopy or robotic) and hemicolectomy with complete mesocolic excision are the treatment of choice for colorectal cancer. The purpose of minimally invasive surgery is to achieve better oncological outcomes while minimizing the incidence of postoperative complications.

The vascularization of the right colon showed considerable variation, *i.e.,* the absence of some branches, supernumerary branches, common trunks and retro-superior courses of the mesenteric vein. To understand vascular variations, a preoperative radiological study is necessary; although there was no concordance between the intraoperative and radiological evaluations, this is a limitation of radiological PII because it is always needed for oncological staging.

It is therefore crucial to increase the knowledge and study of changes in the vascular anatomy of the right colon during surgical resection, particularly during minimally invasive surgery.

We hope that our study can support young surgeons in mastering the learning curve for right colectomy in terms of accurately identifying, exposing, and dissecting right colonic arterial vessels. As previously reported by Wu, knowledge of these variations can help residents reduce the risk of damaging arterial vessels in the right colon and thus avoid troublesome bleeding.

## Data Availability

The dataset generated and/or generated during the current study is not publicly available due to the principle of confidentiality.
